# Cotton metabolism regulatory network: Unraveling key genes and pathways in fiber development and growth regulation

**DOI:** 10.1016/j.xplc.2024.101221

**Published:** 2024-12-12

**Authors:** Zhao Liu, Liqiang Fan, Sheng Shu, Ghulam Qanmber, Eryong Chen, Jinquan Huang, Fuguang Li, Zuoren Yang

**Affiliations:** 1 Zhengzhou Research Base, State Key Laboratory of Cotton Bio-breeding and Integrated Utilization, School of Agricultural Sciences, Zhengzhou University, Zhengzhou 450001, Henan, China; 2 State Key Laboratory of Cotton Bio-breeding and Integrated Utilization, Institute of Cotton Research, Chinese Academy of Agricultural Sciences, Anyang 455000, Henan, China; 3 Xinjiang Key Laboratory of Crop Gene Editing and Germplasm Innovation, Institute of Western Agricultural of CAAS, Changji, Xinjiang 831100, China; 4 Henan Engineering Research Center of Crop Genome Editing, School of Agriculture, Henan Institute of Science and Technology, Xinxiang 453000, Henan, China; 5 National Key Laboratory of Plant Molecular Genetics, CAS Center for Excellence in Molecular Plant Sciences, Shanghai Institute of Plant Physiology and Ecology, Chinese Academy of Sciences, Shanghai 200032, China

**Keywords:** CMRN, cotton, metabolome, transcriptome, *pag1*, metabolic regulatory network

## Abstract

Cotton (*Gossypium hirsutum* L.) is one of the world’s most important commercial crops. However, the dynamics of metabolite abundance and potential regulatory networks throughout its life cycle remain poorly understood. In this study, we developed a cotton metabolism regulatory network (CMRN) that spans various developmental stages and encompasses 2138 metabolites and 90 309 expressed genesin upland cotton. By integrating high-resolution spatiotemporal metabolome and transcriptome data, we identified 1958 differentially accumulated metabolites and 13 597 co-expressed differentially expressed genes between the dwarf mutant *pagoda1* and its wild-type counterpart Zhongmiansuo 24. These metabolites and genes were categorized into seven clusters based on tissue-specific accumulation patterns and gene expression profiles across different developmental stages. Kyoto Encyclopedia of Genes and Genomes enrichment analysis revealed significant differential enrichment in the fatty acid elongation pathway, particularly in fibers. The differential involvement of genes and metabolites in very-long-chain fatty acid (VLCFA) synthesis led to the identification of *GhKCS1b_Dt* as a key gene. Overexpression of *GhKCS1b_Dt* significantly promoted fiber elongation, while its silencing markedly inhibited cotton fiber growth, affirming its positive regulatory role in fiber elongation. This dataset provides a valuable resource for further research into metabolic pathways and gene regulatory networks, offering novel insights for advancing cotton breeding strategies.

## Teaser

This study reports the development of a cotton metabolism regulatory network (CMRN) and the identification of key metabolites and genes involved in various developmental stages. It emphasizes the role of *GhKCS1b_Dt* in fiber elongation and highlights the influence of brassinosteroid metabolism, providing valuable genetic resources for advancing cotton breeding efforts.

## Introduction

Metabolism encompasses a series of chemical reactions that sustain life in living organisms (Danchin, 2017). Bioactive metabolites are categorized as either primary or secondary metabolites based on their functions (Shen et al., 2023). Primary metabolites, including sugars, amino acids, lipids, nucleotides, and vitamins, are crucial energy sources necessary for growth, development, and reproduction (Fiehn, 2002; Park et al., 2022). Secondary metabolites, including antibiotics, toxins, hormones, and pigments, enhance a plant’s adaptability to specific environmental conditions (Fernie and Tohge, 2017). As end products of cellular processes that integrate genetic or environmental information, metabolites are closely linked to the phenotypic status of plants (Wang et al., 2022b). The metabolome serves as a powerful tool for systematically exploring the relationship between genotype and phenotype in plants (Wei et al., 2022). Moreover, the integration of transcriptomics with metabolomics has proven to be a highly effective approach for analyzing changes in gene expression pathways and metabolic processes in plants (Fu et al., 2021; Liu et al., 2023a).

High-throughput sequencing enables simultaneous metabolomics and transcriptomics analyses, identifying key metabolic pathways and their associated genes. This approach can uncover genes and pathways that respond to plant growth, development, stress tolerance, and other traits of interest (Oliver et al., 1998; Yu et al., 2023). For instance, the construction of a metabolic regulation network in rice involved integrating data from 20 samples of each core organ—roots, stems, leaves, and seeds—offering insights into tissue development and metabolic dynamics (Yang et al., 2022; Shen et al., 2023). This integrative approach has also been applied to other plants, such as tomato (*Solanum lycopersicum*) (Zhu et al., 2018), kiwifruit (*Actinidia chinensis*) (Wang et al., 2022a), and cotton (*Gossypium hirsutum*) (Shi et al., 2024). By analyzing high-resolution metabolome and transcriptome data from 20 samples across different tissues, including roots, stems, leaves, flowers, and fruits, and various developmental stages, Li et al. constructed the MicroTom metabolic network, providing a comprehensive view of metabolic regulation throughout the lifespan of tomato (Li et al., 2020). Similarly, metabolomic and transcriptomic analyses in kiwifruit have revealed the regulatory functions of key transcription factors such as *AcERF182* in sugar accumulation and *AcNAC4* in ester biosynthesis (Wang et al., 2022a).

In cotton, metabolomic and transcriptomic analyses have significantly advanced our understanding of essential biological processes, such as drought tolerance and the regulation of flavonoid biosynthesis (Mehari et al., 2021; Shi et al., 2021; Han et al., 2022). Flavonoids are the largest class of secondary metabolites and key components of plant pigments (Liu et al., 2018). Metabolic profiling of fiber pigmentation in green cotton identified critical metabolites primarily enriched in the phenylpropanoid and flavonoid metabolic pathways (Tang et al., 2021). Six flavonoids—(−)-epigallocatechin, apiin, cyanidin-3-O-glucoside, gallocatechin, myricetin, and poncirin—have been reported to promote pigment synthesis in brown cotton fibers (Shi et al., 2024). However, previous studies combining metabolic and transcriptional analyses have primarily focused on single varieties, tissues, or reproductive periods, limiting the availability of comprehensive metabolomic and transcriptomic data across various tissues and throughout the entire growth cycle of cotton. This limitation hinders a thorough understanding of the dynamic changes in metabolites and associated genes throughout the cotton life cycle.

Brassinosteroids (BRs) are essential plant hormones that regulate a wide array of metabolic pathways, including energy production and the synthesis of secondary metabolites like flavonoids and alkaloids (Bajguz and Piotrowska-Niczyporuk, 2014; Qanmber et al., 2024a; Zhang et al., 2024). These hormones enhance the efficient transport of sugars produced during photosynthesis and promote the production of growth factors (Cui et al., 2019; Hu et al., 2021). Additionally, BRs regulate the expression of genes related to nitrogen assimilation, photosynthesis, and carbohydrate metabolism (Hu et al., 2021; An et al., 2023). For instance, the application of 24-epibrassinolide to tomato fruits has been shown to reduce cold injury by modulating the expression of genes associated with photosynthesis and the ethylene biosynthetic pathway (Bai et al., 2021). While substantial evidence supports the role of BRs in controlling metabolite fluctuations, their specific distribution and influence on metabolites within cotton tissues at various developmental stages remain unclear. This ambiguity complicates our understanding of how BRs globally regulate metabolic processes in cotton. The *pagoda1* (*pag1*) mutant, encoded by the *GhPAG1* gene—an ortholog of CYP734A1—is crucial for BR biosynthesis and homeostasis. The *pag1* mutant exhibits a range of distinctive phenotypic traits, including dwarfism characterized by shortened stems and internodes, delayed senescence, and wrinkled leaves with short petioles. It also produces short flowers with reduced viability and small bolls, locules, and seeds. Additionally, this mutant shows reduced fiber length and strength due to significant inhibition of cell elongation and expansion (Yang et al., 2014). Therefore, this mutant serves as an excellent model for studying BR regulation in cotton growth and development.

In this study, we systematically integrated metabolomic and transcriptomic data to investigate the metabolic profile of cotton throughout its entire life cycle. We harvested 30 different tissues representing roots, stems, leaves, flowers, ovules, and fibers from Zhongmiansuo 24 (ZM24) and *pag1* cotton plants across seven critical developmental stages, including 5-day-old roots, 15-day-old roots, stems, and leaves, 35-day-old stems and leaves, 70-day-old stems, leaves, and flowers, as well as ovules and fibers at 5, 10, and 20 days post-anthesis (DPA). We developed a comprehensive cotton metabolism regulatory network (CMRN) to study the metabolomic and transcriptomic changes occurring in these critical tissues and growth stages. This network has enabled us to identify key metabolites involved in the biosynthetic pathway of fiber development and genes associated with metabolism and signal transduction. These findings provide valuable genetic resources for further research into cotton fiber development.

## Results

### Metabolite accumulation patterns during cotton growth and development

**Figure 1:**
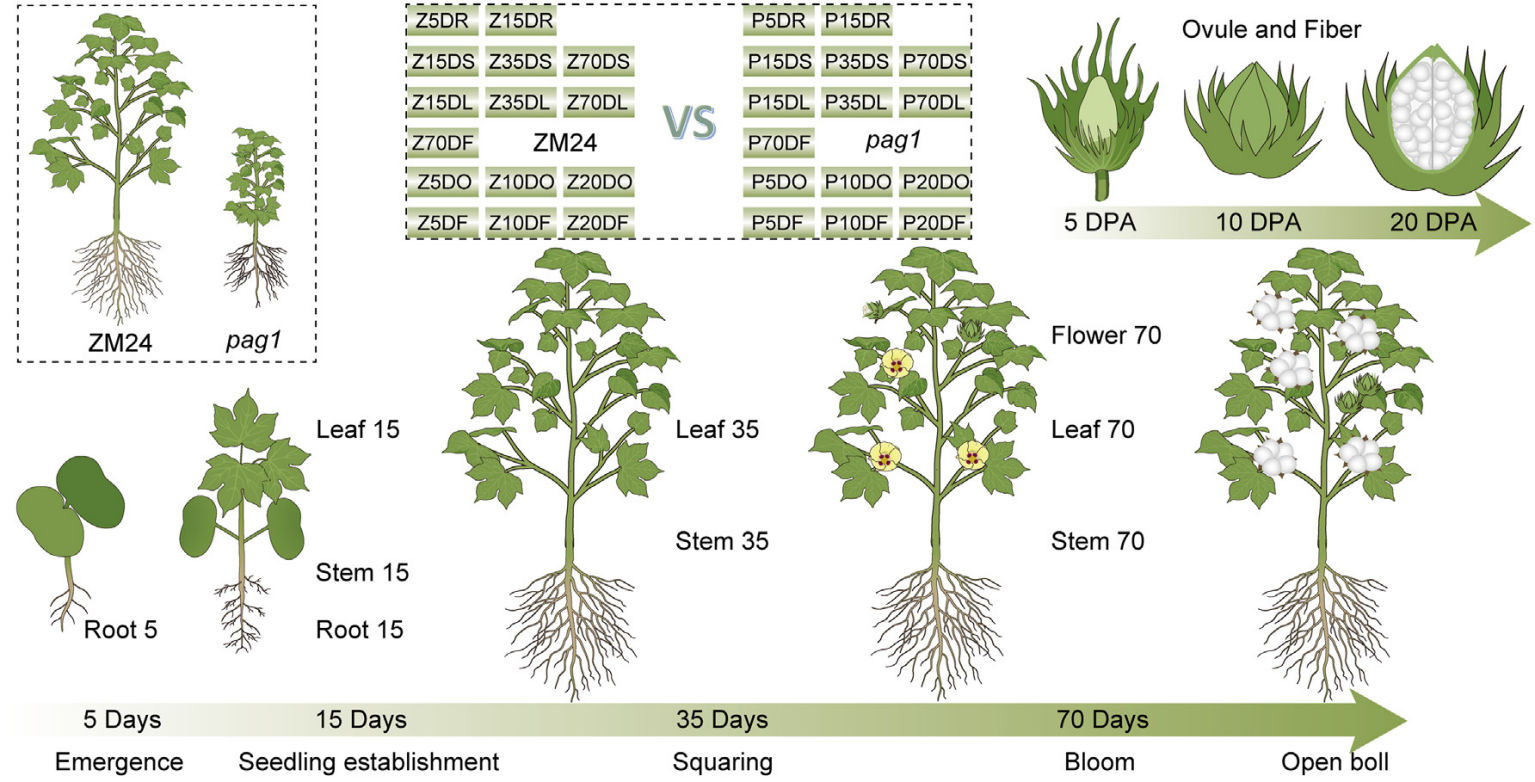
Schematic representation of the CMRN study design. Ninety samples from two materials, the *G. hirsutum* cultivar ZM24 and the BR-deficient mutant *pag1*, were collected at seven key developmental stages for metabolic profiling and gene expression analysis. The samples included roots (5 and 15 days post-seeding), stems (15, 35, and 70 days post-seeding), leaves (15, 35, and 70 days after emergence of the first true leaf), flowers (70 days after seeding), and ovules and fibers (5, 10, and 20 days post-anthesis).

**Figure 2:**
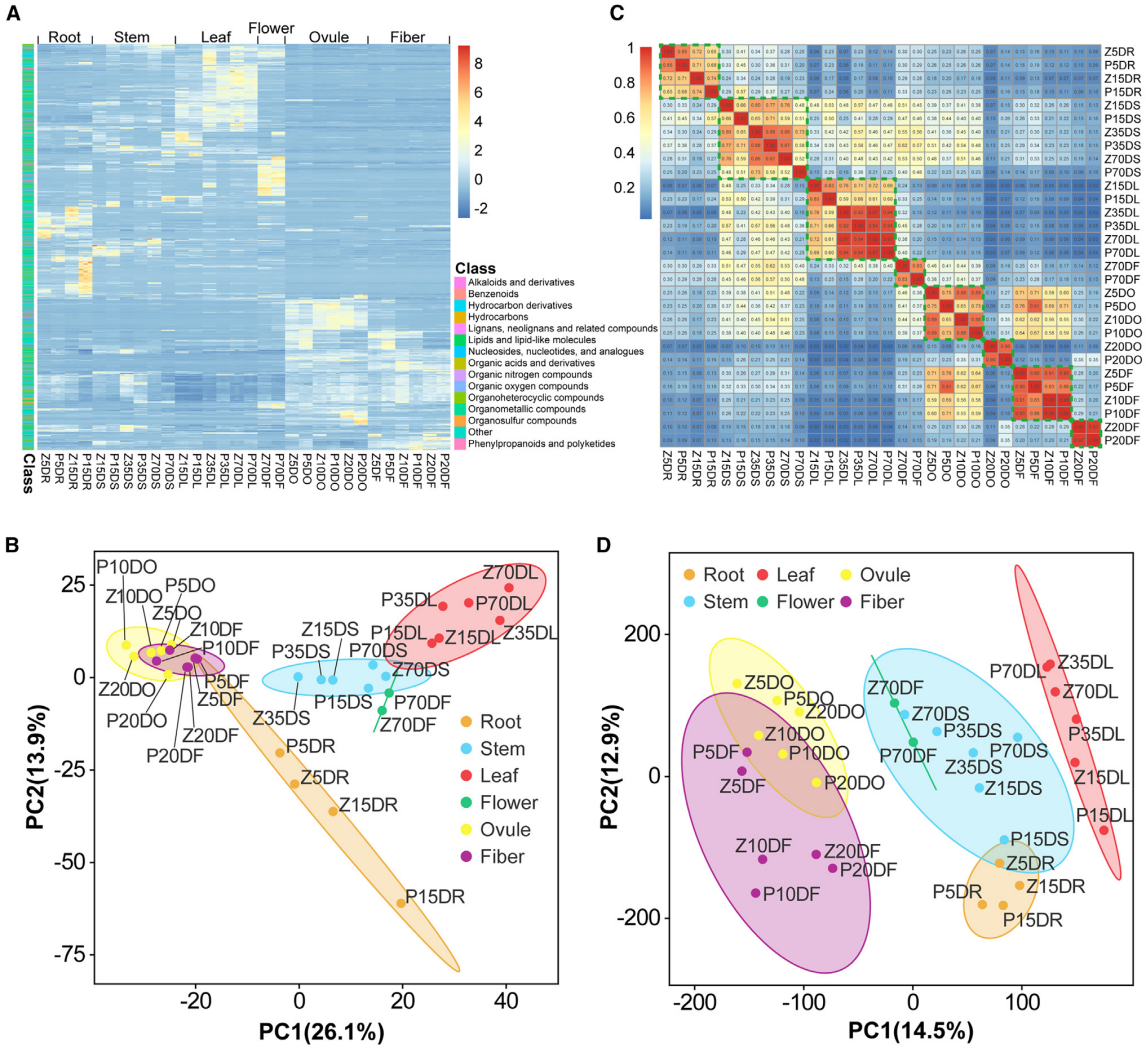
Summary of metabolome and transcriptome data of the CMRN. **(A)** Overview of the 2138 annotated metabolites identified from the 90 cotton samples. **(B)** PCA of metabolome data derived from the 90 cotton samples. **(C)** Hierarchical clustering analysis of expression profiles for 90 309 genes across the 90 cotton samples. **(D)** PCA of the transcriptome data from the 90 cotton samples. For the metabolome data **(A)**, each row represents a metabolite with *Z*-scores standardized between −2 and 8. For the transcriptome data **(C)**, the color scale ranging from 0 to 1 represents Spearman’s correlation coefficients.

To study metabolite accumulation patterns across seven key developmental stages (90 samples) in two cotton materials, *G. hirsutum* cultivar ZM24 and the BR-deficient mutant *pag1*, we conducted metabolic profiling (Figure 1). Compared to the wild type, the *pag1* mutant exhibited dwarfism and reduced lengths of leaves, seeds, and fibers due to reduced BR (brassinolide [BL] and castasterone [CS]) contents (Supplemental Figure 1). A total of 2138 distinct annotated metabolites were identified in at least one tissue. These included 18 alkaloids and derivatives, 113 benzenoids, 54 lipids, one hydrocarbon derivative, 10 hydrocarbons, four lignans, neolignans, and related compounds, 280 lipids and lipid-like molecules, 40 nucleosides, nucleotides, and analogs, 128 organic acids and derivatives, 31 organic nitrogen compounds, 83 organic oxygen compounds, 128 organoheterocyclic compounds, one organometallic compound, two organosulfur compounds, 135 phenylpropanoids and polyketides, and 1164 additional compounds that did not fit into these 15 main classes (Figure 2A; Supplemental Table 1). Hierarchical clustering analysis of the 2138 metabolites from different tissues revealed that they could be divided into six major groups associated with root, stem, leaf, flower, ovule, and fiber tissues (Figure 2A). This suggests that while similar metabolic patterns occur in the same tissue types across different materials and developmental stages, significant differences exist among different tissues.

In addition, hierarchical clustering of the 2138 metabolites from different tissues was performed using principal-component analysis (PCA), which classified the tissues into six groups, each associated with a specific tissue type (Figure 2B). Notably, ovules and fibers were clustered nearby, suggesting that their metabolic profiles were more similar to each other than to other tissues. Overall, these results indicate that metabolite accumulation patterns during cotton development are highly tissue-specific.

### Transcriptional regulation during cotton growth and development

We investigated transcriptional regulation during cotton growth and development by constructing dynamic transcriptome profiles for all tissues of two cotton materials. Approximately 599.90 Gb of clean data were obtained, and the quality of the data and their mapping to the reference genome are shown in Supplemental Table 2. A total of 90 309 genes (both annotated and unannotated) were found to be expressed in at least one tissue by aligning the transcriptome data to the reference genome.

Correlation matrix analysis of these 90 309 genes revealed that global gene expression patterns in ZM24 and *pag1* at different developmental stages were tissue-specific (Figure 2C; Supplemental Table 3). Notably, ovules and fibers at 20 DPA exhibited significant differences in gene expression patterns compared to those at 5 and 10 DPA, suggesting substantial changes in gene expression at later stages of development. Consistent with the metabolome results, PCA of the transcriptome data revealed clustering of the same tissue types from different materials at different stages (Figure 2D). These findings indicate significant tissue specificity in both gene expression and metabolite accumulation.

### Co-regulation of the cotton metabolome and transcriptome across various tissues

**Figure 3:**
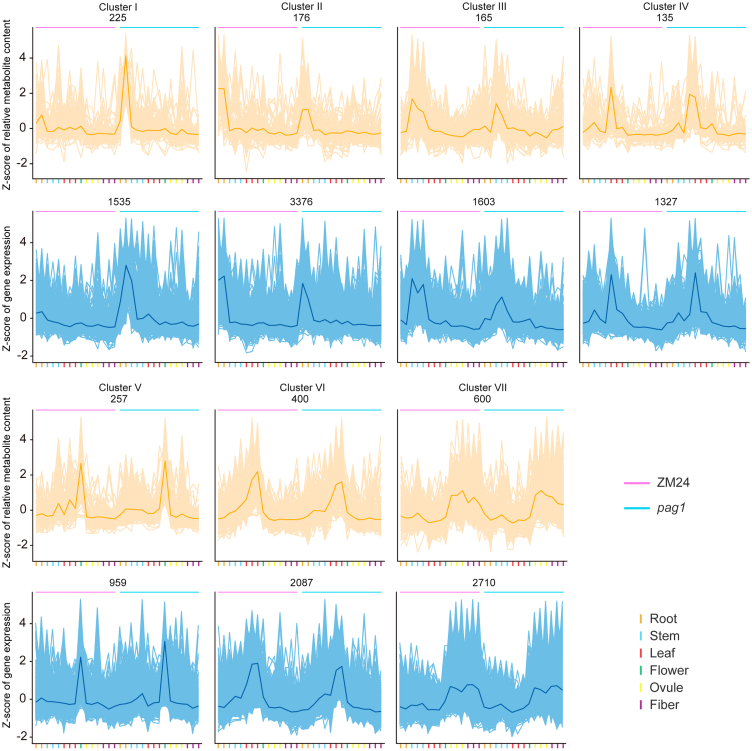
Dynamics of metabolite content and gene expression during the cotton growth cycle. K-means clustering categorized the expression profiles of the cotton metabolome (orange) and transcriptome (blue) into seven clusters. The x axis represents the 90 samples from seven key developmental stages, while the y axis shows the *Z*-scores standardized for each metabolite (orange) and gene (blue). Each box displays the total number of metabolites and genes (e.g., 225 metabolites and 1535 genes for cluster I) within all 90 samples for each cluster.

We performed co-expression analysis using metabolomic and gene expression profiling data to explore genetic and metabolic transformations in different tissues of both BR-deficient and normal cotton plants during their growth and development. Initially, we screened 1958 metabolites for significant differences in at least one tissue between the two materials. Subsequently, a rigorous multiple test correction (r > 0.8) identified 13 597 genes significantly co-regulated with at least one differential metabolite. PCA of these 1958 metabolites and 13 597 genes revealed higher tissue specificity (Supplemental Figures 2A and 2B), suggesting that major metabolic pathways and gene expression patterns exhibit consistent dynamics across tissues during the cotton growth cycle. These metabolites and genes were subsequently classified into seven co-response clusters based on Pearson correlation coefficients, displaying uniform and clear abundance patterns during cotton development (Figure 3; Table 1). This indicates that while gene expression patterns and metabolic pathways differ significantly in the same tissues of ZM24 and *pag1*, the overall dynamics are consistent throughout the growth cycle.

**Table 1:** Distribution of metabolites and genes identified in this study across different clusters.

	Cluster I	Cluster II	Cluster III	Cluster IV	Cluster V	Cluster VI	Cluster VII
Compounds	225	176	165	135	257	400	600
Genes	1535	3376	1603	1327	959	2087	2710
Alkaloids and derivatives	0	1	2	1	1	5	6
Benzenoids	8	6	11	7	14	23	36
Hydrocarbon derivatives	0	0	0	0	1	0	0
Hydrocarbons	0	0	0	0	0	5	4
Lignans, neolignans, and related compounds	1	1	0	0	0	1	1
Lipids and lipid-like molecules	20	20	38	21	16	67	83
Nucleosides, nucleotides, and analogs	3	2	0	1	6	4	18
Organic acids and derivatives	21	9	10	4	10	21	42
Organic nitrogen compounds	4	2	6	3	4	1	10
Organic oxygen compounds	8	10	2	8	9	16	23
Organoheterocyclic compounds	17	9	11	9	17	26	31
Organosulfur compounds	0	1	0	0	0	0	0
Other	135	103	80	78	143	209	306
Phenylpropanoids and polyketides	8	12	5	3	36	22	40

**Figure 4:**
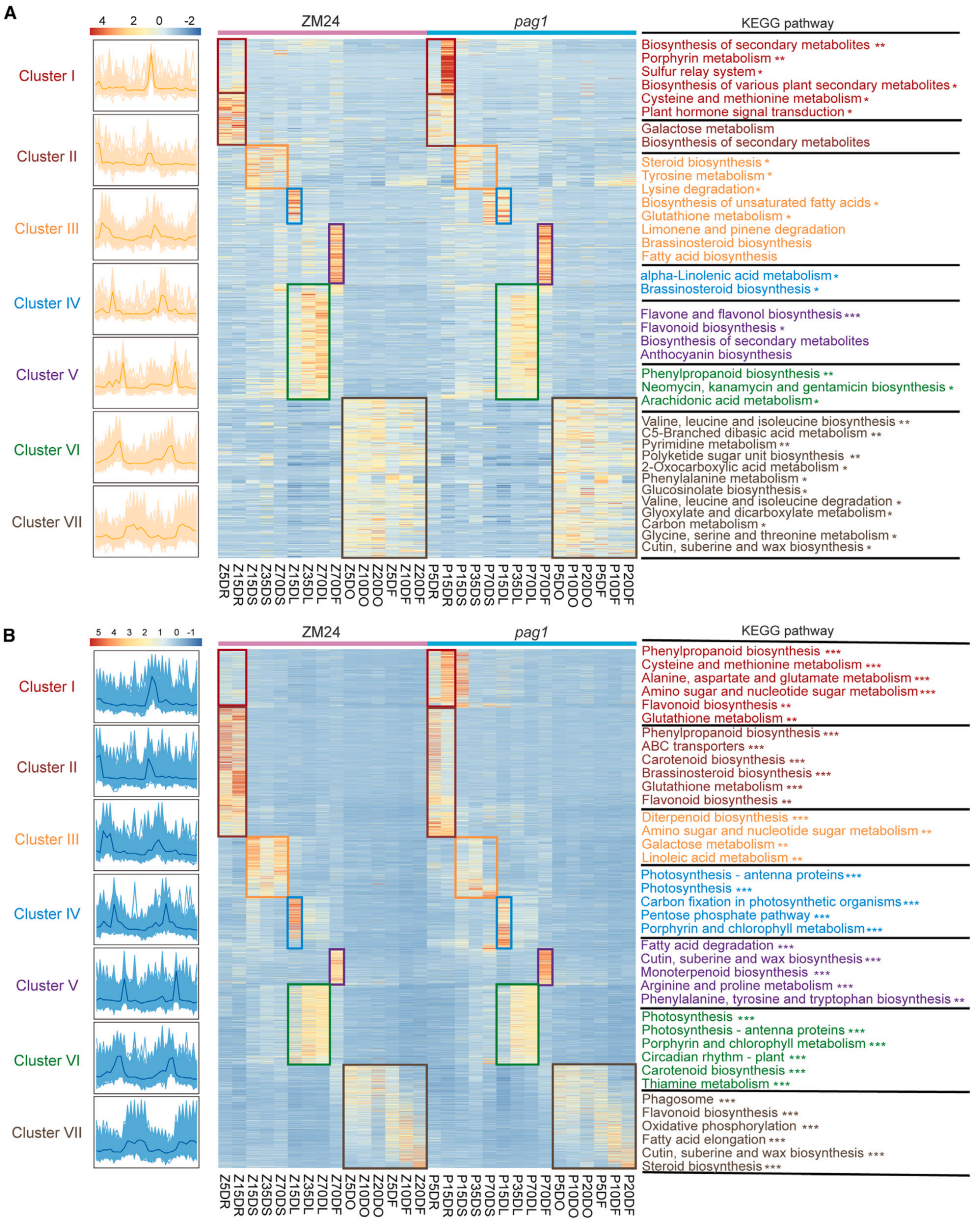
Functional characteristics of metabolite content and gene expression throughout the cotton growth cycle. Seven clusters show the tissue specificity of BR in both normal and deficient conditions through co-expression analysis. The left and middle sections show the co-expression patterns of metabolite accumulation **(A)** and gene expression **(B)** across the seven clusters. Metabolites **(A)** and genes **(B)** associated with similar biological processes are grouped by the degree of enrichment, which is indicated on the right side. The seven bars of different colors represent the metabolites **(A)** and genes **(B)** enriched in each of the seven clusters.

To provide a comprehensive overview of metabolite content and gene expression across 30 tissues, we generated a *Z*-score normalized expression heatmap, clustered by metabolites and genes. Our analysis of these seven clusters identified 225 metabolites that accumulated significantly less in the root tissues of ZM24 (Z5DR, Z15DR) compared to *pag1* (P5DR, P15DR) (cluster I). Conversely, 176 metabolites showed the opposite accumulation pattern (cluster II) (Figure 3; Table 1). The other five clusters showed differential metabolite accumulation unique to specific tissues in ZM24 and *pag1*: cluster III (165 metabolites) corresponded to stem tissues, clusters IV (135 metabolites) and VI (400 metabolites) to leaf tissues, cluster V (257 metabolites) to flower tissues, and cluster VII (600 metabolites) to ovule and fiber tissues (Figure 3; Table 1). Kyoto Encyclopedia of Genes and Genomes (KEGG) pathway enrichment analysis of differential metabolites in these co-expression clusters revealed distinct patterns, despite the presence of multiple metabolic pathways within individual clusters (Figure 4A; Supplemental Table 4). For example, metabolites involved in the biosynthesis of secondary metabolites were predominantly enriched in clusters I (root), II (root), and V (flower). Metabolites implicated in BR biosynthesis were enriched in clusters III (stem) and IV (leaf), those in phenylpropanoid biosynthesis in cluster VI (leaf), and those in phenylalanine metabolism in cluster VII (ovule and fiber).

Similarly, the 13 597 genes were clustered into seven clusters, each exhibiting a distinct expression pattern in different tissues of ZM24 and *pag1* (Figure 4B; Table 1). KEGG enrichment analysis revealed that genes involved in phenylpropanoid biosynthesis were enriched in clusters I and II (root), whereas those involved in diterpenoid biosynthesis were enriched in cluster III (stem). Additionally, genes related to photosynthesis, including photosynthesis antenna proteins, photosynthesis pathways, and porphyrin and chlorophyll metabolism, were enriched in clusters IV and VI (leaf). Genes involved in fatty acid degradation were enriched in cluster V (flower), and those involved in fatty acid elongation were enriched in cluster VII (ovule and fiber) (Figure 4B; Supplemental Table 5).

A correlation analysis between transcriptional and metabolic pathways in different tissues further elucidated the dynamic relationship between transcript levels and metabolite content in *pag1* compared with ZM24 (Supplemental Figure 3; Supplemental Table 6). In roots (clusters I and II) and flowers (cluster V), significant changes in metabolite content within the biosynthesis of secondary metabolites were primarily associated with changes in gene expression within transcriptional pathways, including phenylpropanoid biosynthesis, and cysteine and fatty acid degradation. In stems (cluster III), variations in gene expression within the diterpenoid biosynthesis and amino sugar and nucleotide sugar metabolism pathways affected the number of metabolites in eight pathways, including fatty acid biosynthesis. In leaves (clusters IV and VI), pathways associated with photosynthesis, such as photosynthesis antenna proteins, photosynthesis, carbon fixation in photosynthetic organisms, porphyrin and chlorophyll metabolism, underwent substantial changes, affecting the contents of metabolites in pathways such as alpha-linolenic acid metabolism and BR biosynthesis. In ovules and fibers (cluster VII), strong correlations were observed between six gene pathways, including fatty acid elongation, and eight metabolic pathways, such as phenylalanine metabolism, potentially affecting fiber development. This comprehensive metabolic and transcriptomic dataset not only enhances our understanding of BR-related metabolic pathways but also aids in the identification of candidate genes across various cotton tissues throughout its life cycle.

### Genetic basis of changes in BR-related metabolites in ZM24 and *pag1*

**Figure 5:**
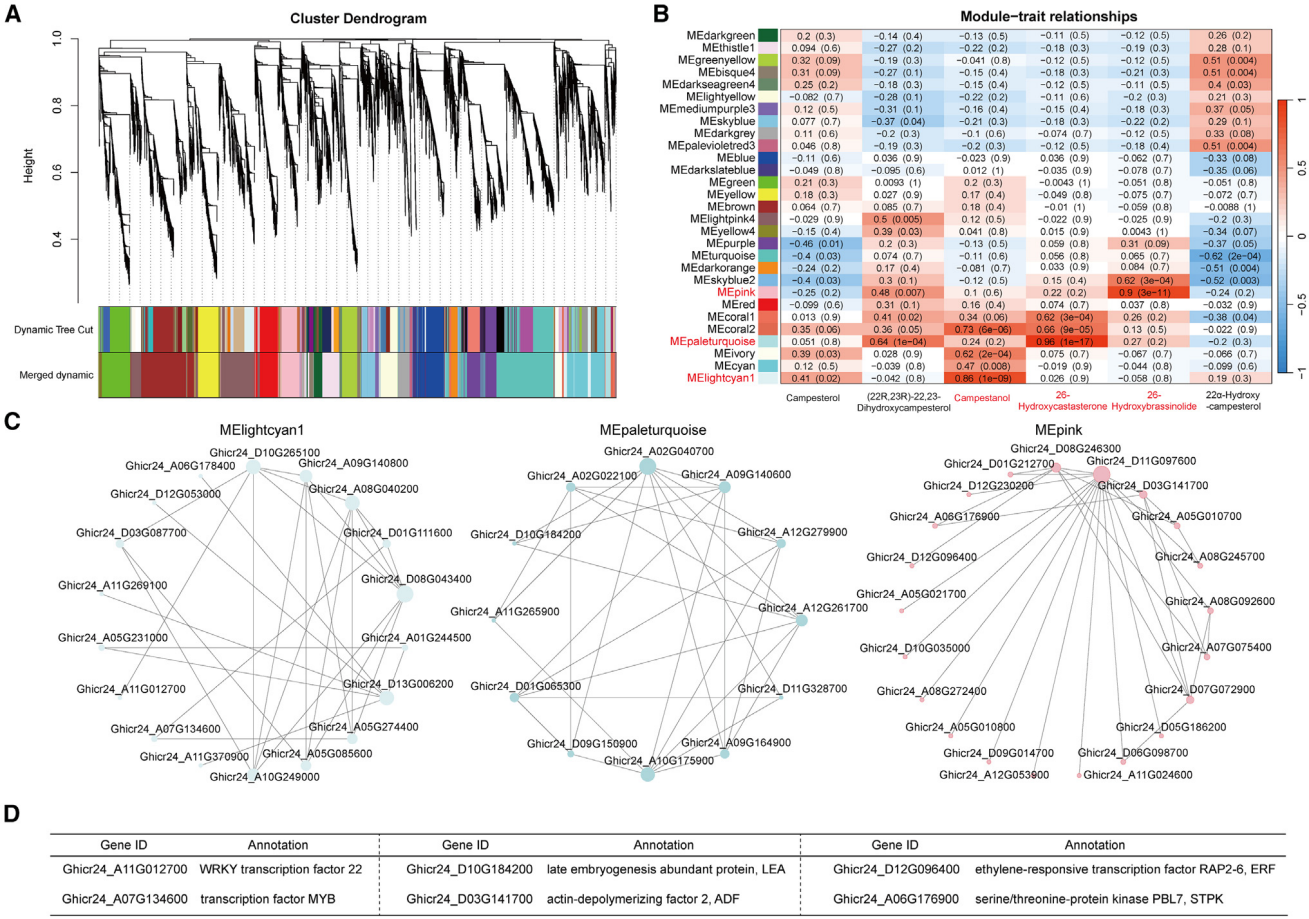
WGCNA results for 13 597 genes co-regulated with at least one metabolite. Transcriptome and metabolic correlation analysis of different tissues at various growth stages in cotton. **(A)** A hierarchical clustering tree (cluster dendrogram) showing 29 expression modules, each labeled with a different color. **(B)** Module–BR synthetic pathway metabolite relationship analysis. Inside each box, the value represents Pearson’s correlation coefficient between the module and BR synthesis pathway metabolites; the number in parentheses indicates the *P* value. The color scale on the right shows the degree of correlation, with red indicating a positive correlation and blue indicating a negative correlation. **(C)** A directed interaction network diagram illustrating relationships between DEGs and key metabolites including campestanol (MElightcyan 1 module), 26-hydroxycastasterone (MEpaleturquoise module), and 26-hydroxybrassinolide (MEpink module). **(D)** Functional annotation of six hub genes.

To further understand the regulation of metabolic changes in ZM24 and *pag1* throughout their entire life cycle, we performed a weighted gene co-expression network analysis to investigate the co-expression networks of 13 597 genes. This analysis identified 29 co-expression modules based on similar expression patterns (Figure 5A). The heatmap of module–trait correlations (Figure 5B) revealed that the lightcyan1 module (181 genes) was correlated with campestanol, the paleturquoise module (144 genes) with hydroxycastasterone, and the pink module (576 genes) with hydroxybrassinolide, which are key compounds in BL biosynthesis. These results indicate that genes in these three modules are primarily associated with changes in BR metabolism throughout the cotton life cycle.

The first step in the BR synthesis pathway involves the conversion of campesterol to campestanol, catalyzed by the enzyme de-etiolated2 (Noguchi et al., 1999). In the lightcyan1 module, 18 hub genes were identified with an edge weight value ≥ 0.35 (Figure 5C), including WRKY transcription factor 22 (Ghicr24_A11G012700) and a MYB transcription factor (Ghicr24_A07G134600) (Supplemental Figure 4A; Supplemental Table 7). In the paleturquoise module, 12 hub genes associated with CS—a biologically active substance similar to BL and a direct precursor to BL (Yokota et al., 1982)—were identified with an edge weight value ≥0.45 (Figure 5C), such as the late embryogenesis abundant protein (Ghicr24_D10G184200) (Supplemental Figure 4B; Supplemental Table 7). BL, the most active and well-studied BR (Grove et al., 1979), had 21 hub genes in the pink module with an edge weight value ≥0.6 (Figure 5C), including actin-depolymerizing factor 2 (Ghicr24_D03G141700), ethylene-responsive transcription factor (Ghicr24_D12G096400), and serine/threonine-protein kinase (Ghicr24_A06G176900) (Supplemental Figure 4C; Supplemental Table 7).

### Tissue-specific BR biosynthesis and signaling genes in cotton

We then comprehensively examined the expression profiles of BR-related genes in different tissues throughout the entire life cycle of cotton (Supplemental Figure 5; Supplemental Table 8). We found that genes involved in BR biosynthesis and signaling pathways have less impact on roots. Stem length is a key determinant of cotton plant height. Our previous study has demonstrated that *GhPAS1* positively regulates root development and stem length (Wu et al., 2021a), whereas *GhPAG1* negatively influences these traits (Chen et al., 2019). Under BR-deficient conditions, cotton exhibits dwarf phenotypes, characterized by reduced leaf size and shortened stems. Both *GhEXO2* (Li et al., 2023) and *GhEXO7* (Li et al., 2021) positively regulate leaf size and stem length in cotton, whereas *GhPAG1* (Yang et al., 2014) has a negative role. Research on genes related to BR regulation of cotton fibers is a hot topic, with most genes being positively regulated, such as *GhBES1.4*, *GhKCS10* (Yang et al., 2023), *GhKRP6* (Gu et al., 2023), *GhbHLH282* (Lu et al., 2018), *GhEB1C1* (Mao et al., 2024), *GhSMO2* (Liu et al., 2023b), *Gh14-3-3* (Tian and Zhang, 2021), *GhSMT2* (Luo et al., 2007), *GhBRI1* (Sun et al., 2015), *GhDET2* (Luo et al., 2007), and *GhDET3* (Xiao et al., 2008). Conversely, genes such as *GhSK13* (Wang et al., 2020), *GhPAG1* (Yang et al., 2014), and *GhBIN2* (Sun and Allen, 2005), are negatively regulated. We have aligned the sequences of these genes with those of the corresponding tissues analyzed in this study and displayed their expression profiles, offering a valuable resource for further BR-related studies (Supplemental Figure 6; Supplemental Table 8).

### Impact of BR deficiency on *KCS* gene expression and VLCFA synthesis in cotton fibers

**Figure 6:**
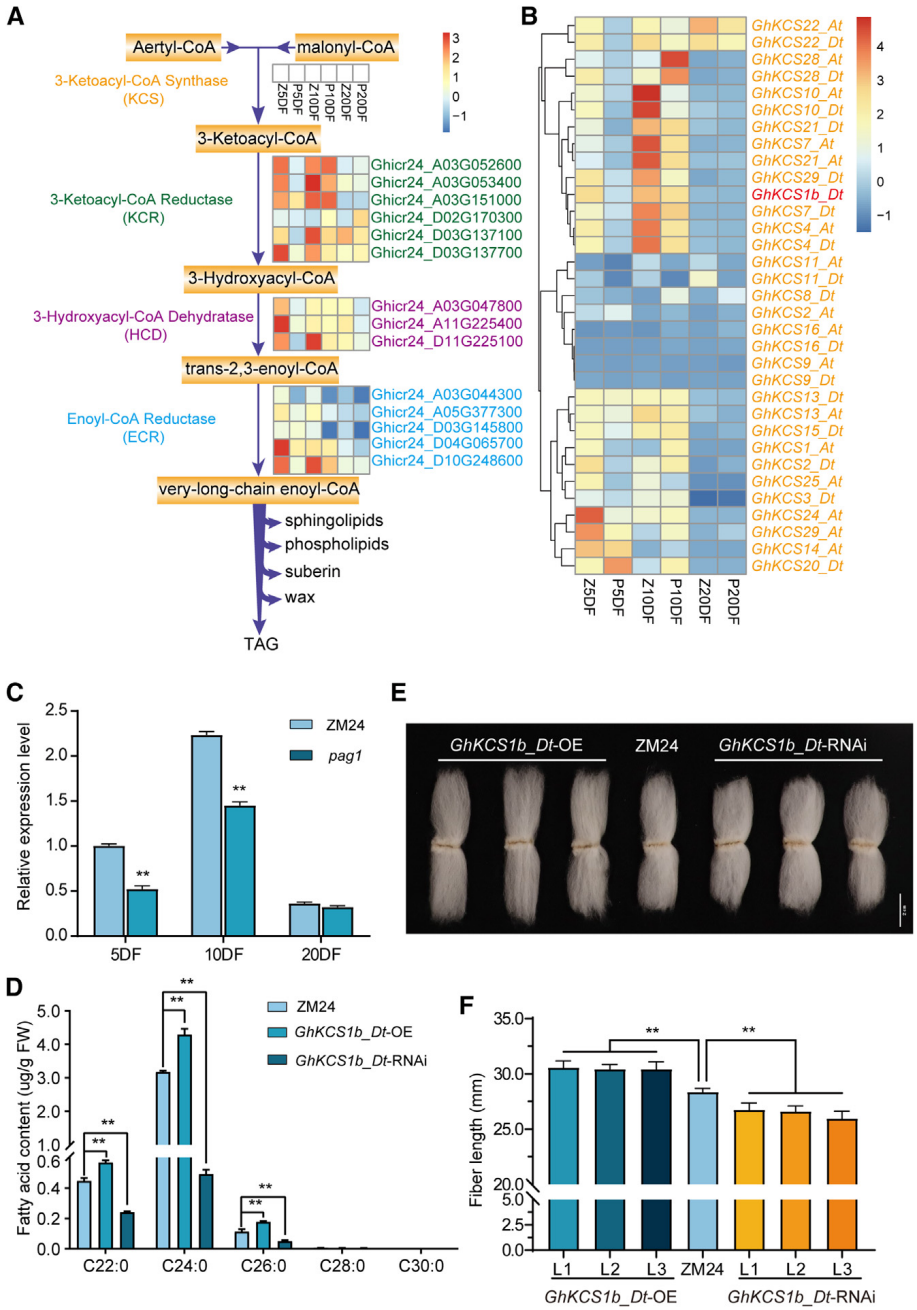
*KCS1b_Dt* positively regulates cotton fiber elongation. **(A)** Differential expression profiles of genes related to fatty acid synthesis in ZM24 and *pag1* fibers. **(B)** Heatmap showing the expression patterns of 33 *KCS* genes in cotton fibers at three developmental stages in ZM24 and *pag1*. **(C)** Expression levels of the *GhKCS1b_Dt* gene in ZM24 and *pag1* fibers at 5, 10, and 20 DPA. **(D)** Fatty acid content in 10 DPA fibers of ZM24, *GhKCS1b_Dt*-OE, and *GhKCS1b_Dt*-RNAi lines. The data were collected from three independent experiments, with 30 fibers measured per treatment. Data are presented as the mean ± SD of three experiments. **(E)** Phenotypes of *GhKCS1b_Dt*-OE/*GhKCS1b_Dt*-RNAi and ZM24 mature fibers. Bar: 2 cm. **(F)** Lengths of *GhKCS1b_Dt*-OE/*GhKCS1b_Dt*-RNAi and ZM24 mature fibers. Error bars represent the SD from 30 different mature fibers of *GhKCS1b_Dt*-OE/*GhKCS1b_Dt*-RNAi and ZM24, respectively. All data reported here were obtained from three independent experiments, with 30 mature fibers measured for each treatment, and are presented as mean ± SD of triplicate experiments. Significant differences compared with ZM24 were determined using a one-way ANOVA combined with a Student’s *t*-test (∗∗*P* < 0.01 by *t*-test).

Previous studies have shown that BR deficiency reduces *GhKCS* expression, leading to lower levels of saturated VLCFAs in *pag1* fibers (Yang et al., 2014, 2023). Our current study reveals significant differences in metabolite content between ZM24 and *pag1* cotton. Specifically, the KEGG pathway in cluster III was significantly enriched for the biosynthesis of unsaturated fatty acids and fatty acid biosynthesis pathways (Figure 4A; Supplemental Table 4). Moreover, differentially expressed genes (DEGs) in clusters V and VII were significantly enriched in KEGG pathways related to fatty acid degradation and elongation, respectively (Figure 4B; Supplemental Table 5). This indicates that VLCFA synthesis is closely linked to fatty acid synthesis, degradation, and elongation. VLCFA synthesis involves four enzymes: β-ketoacyl-coenzyme A (CoA) synthase (KCS), β-ketoacyl-CoA reductase (KCR), β-hydroxyacyl-CoA dehydratase (HCD), and enoyl-CoA reductase (ECR) (Yang et al., 2023). In this study, a total of 47 genes related to these enzymes were identified in VLCFA-associated pathways. Among these, 26, 17, and 6 genes exhibited higher expression levels in ZM24 fibers than in *pag1* fibers at 5, 10, and 20 DPA, respectively (Figures 6A and 6B). Among these enzymes, KCS is considered the rate-limiting enzyme for fatty acid elongation. Our previous study demonstrated that 35 *GhKCS* genes were expressed in ZM24 fibers at four stages (5 DPA fiber [5DF], 10DF, 20DF, 25DF) (Yang et al., 2023), with *GhKCS1b_Dt* showing the highest expression at the 10DF stage, followed by a significant decrease from the Z10DF to the Z20DF and ZM24 25 DPA (Z25DF) stages (Supplemental Figure 7). Unfortunately, no further studies were performed on this gene. Using the CMRN dataset, we identified 33 fiber-specific *GhKCS* genes differentially expressed at three stages (5DF, 10DF, 20DF) between ZM24 and *pag1* fibers. Notably, *GhKCS1b_Dt* expression decreased significantly at 5DF and 10DF in *pag1* compared to ZM24 fibers (Figure 6B). qRT–PCR analysis confirmed that *GhKCS1b_Dt* expression levels decreased by 46.5% and 35.1%, respectively, at 5DF and 10DF in *pag1* compared to ZM24 (Figure 6C). To investigate the role of *GhKCS1b_Dt* in cotton fiber development, we generated *GhKCS1b_Dt* overexpression (*GhKCS1b_Dt*-OE) and RNA-interference (*GhKCS1b_Dt*-RNAi) cotton lines. The OE and knockdown of *GhKCS1b_Dt* in these lines were confirmed at the DNA, RNA, and protein levels (Supplemental Figures 8A–8E). The levels of saturated VLCFAs, including C22:0, C24:0, and C26:0, were significantly increased by 26.91%, 35.29%, and 57.07%, respectively, in *GhKCS1b_Dt*-OE lines compared to ZM24, whereas these levels were significantly reduced by 46.09%, 84.56%, and 55.04% in *GhKCS1b_Dt*-RNAi lines (Figure 6D). Fiber length increased by 7.97% in *GhKCS1b_Dt*-OE lines compared to ZM24 but decreased by 6.78% in *GhKCS1b_Dt*-RNAi lines (Figures 6E and 6F). Furthermore, *in vitro* ovule culture experiments demonstrated that the exogenous application of C24:0 could partially restore the short-fiber phenotype of *GhKCS1b_Dt*-RNAi lines, improving fiber length by 24.41% (Supplemental Figures 9A and 9B). These results suggest that *GhKCS1b_Dt* positively regulates cotton fiber development.

## Discussion

BR is an essential hormone for cotton growth and development. Analyzing the metabolic profile of the BR-deficient mutant *pag1* throughout its life cycle provides an ideal model for studying BR regulation in cotton. We systematically integrated the metabolome and transcriptome from 90 samples covering major tissues and growth stages of the *G. hirsutum* cultivar ZM24 and the BR-deficient mutant *pag1*. The CMRN dataset generated from this study includes data on 2138 metabolites and 90 309 expressed genes detected in at least one tissue or stage. Based on this dataset, we identified 1958 metabolites that differed significantly in at least one tissue between the two cotton materials and grouped them into seven clusters. Additionally, 13 597 genes that co-expressed (r > 0.8) with at least one of these 1958 metabolites were identified.

Our CMRN dataset is a valuable addition to existing multi-omics resources for cotton. Previous metabolic and transcriptional studies have primarily focused on single varieties, specific tissues, or distinct reproductive periods, often resulting in limited comprehensive data across various tissues throughout the entire growth cycle of cotton. This limitation has posed challenges in analyzing regulatory networks. Our CMRN dataset covers most tissues and developmental stages, providing a high-resolution map of metabolic and transcriptional dynamics throughout the cotton growth cycle (Figure 2A; Table 1). Utilizing the CMRN dataset, we identified significant metabolic changes between the different tissues of ZM24 and *pag1* (Figure 3). These transcriptional changes underlying significant metabolic shifts are captured by the CMRN, offering a comprehensive resource for investigating metabolic regulation.

KEGG analysis delineated the distinct biological functions of different tissues under BR-deficient conditions, revealing seven clusters that characterize changes in the metabolome and transcriptome. Wang et al. (2023) demonstrated that BR modulates sulfur metabolism by inhibiting glucosinolate accumulation and enhancing the biosynthesis of primary sulfur metabolites, such as cysteine and glutathione, in both *Arabidopsis* and *Brassica* crops. This modulation promotes plant growth by fine-tuning secondary and primary sulfur metabolism. In roots, changes in metabolite content within the biosynthesis pathway of secondary metabolites were mainly associated with alterations in gene expression within transcriptional pathways, such as phenylpropanoid biosynthesis, cysteine, and glutathione metabolism (Supplemental Figure 3). The MYB transcription factor, a key regulator of phenylpropanoid metabolism, promotes the biosynthesis of phenylalanine to provide more aromatic amino acids for secondary metabolism, potentially contributing to the short root phenotype observed in the *pag1* mutant (Supplemental Figure 1D). BR biosynthesis promotes cell elongation and division and affects xylem differentiation. In stems, variations in metabolite content in the BR biosynthesis pathway could impact the plant height of the *pag1* mutant. Several studies have shown that BRs promote photosynthesis (Xia et al., 2009; Wang et al., 2012). In leaves, significant changes in metabolite levels and gene expression were mainly observed in BR biosynthesis- and photosynthesis-related pathways (photosynthesis antenna proteins, photosynthesis, carbon fixation in photosynthetic organisms, and porphyrin and chlorophyll metabolism) (Supplemental Figure 3), potentially leading to the observed wrinkled leaves with short petioles in *pag1* (Supplemental Figure 1B). Significant changes in ovule and fiber tissues were primarily enriched in pathways such as flavonoid biosynthesis, fatty acid elongation, and steroid biosynthesis (Figure 4B; Supplemental Table 5). Peng et al. noted that flavonoid biosynthesis is involved in the pigmentation of naturally brown-colored cotton fibers (Peng et al., 2020). Similarly, fatty acid elongation is crucial for forming VLCFAs, an essential component of cotton fiber elongation (Yang et al., 2023). Additionally, steroid biosynthesis, which modulates plant hormones, particularly BRs, influences fiber elongation (Yang et al., 2014, 2023). These metabolic changes likely contribute to the significantly shorter fibers observed in the *pag1* mutant.

VLCFAs are synthesized in the endoplasmic reticulum by four enzymes: KCS, KCR, HCD, and ECR (Haslam and Kunst, 2013). Using the CMRN dataset, we identified 33 *GhKCS*, 6 *GhKCR*, 3 *GhHCD*, and 5 *GhECR* DEGs at various stages of fiber development (5DF, 10DF, 20DF) between ZM24 and *pag1* (Figures 6A and 6B), showing an overall downregulation trend in *pag1* compared to ZM24. Meng et al. reported that three *GhKCR* genes in cotton, notably *GhKCR1* and *GhKCR2*, were highly expressed during the elongation stage of cotton fibers, with their expression levels peaking at 5 DPA in fiber cells. Inhibition of *GhKCR* gene expression has been shown to hinder sphingolipid synthesis and inhibit fiber elongation (Meng et al., 2022). In this study, the expression of the four *GhKCR* genes at 5DF and 10DF was significantly reduced in *pag1* compared with ZM24 (Figure 6A), highlighting their potential importance in fiber development. Previous studies have indicated that VLCFA content was significantly lower in *pag1* compared to ZM24, with several *GhKCS* genes significantly downregulated in 10 DPA fibers (Yang et al., 2023), particularly *GhKCS10_At, which showed a dramatic reduction of 87.72%, a finding* also verified in this study (Figure 6B). Besides *GhKCS10_At*, significant changes in *GhKCS1b_Dt* expression in 10 DPA fibers were previously reported (Yang et al., 2023), which aligns with the differential expression observed in 5DF and 10DF fibers between ZM24 and *pag1* (Figure 6B). To validate the transcriptome data, qRT–PCR analysis showed that *GhKCS1b_Dt* was downregulated by 46.5% and 35.1% in 5DF and 10DF fibers of *pag1*, respectively (Figure 6C). In ZM24 fibers, genes from the *GhKCS family* were found to be overexpressed compared to those in *pag1*, and BR notably affected the *GhKCS10_At* expression through the regulation of *GhBES1.4*, thereby affecting VLCFA synthesis and fiber elongation (Yang et al., 2023; Qanmber et al., 2024b). Similar to other members of the *GhKCS* family, *GhKCS1b_Dt* exhibited high expression in the 5DF and 10DF fibers of ZM24 compared to those of *pag1* (Figure 6B). Transgenic cotton plants overexpressing *GhKCS1b_Dt* showed a 7.97% increase in fiber length, whereas RNAi lines exhibited a 6.78% decrease (Figures 6E and 6F). These results suggest that *GhKCS1b_Dt* positively regulates fiber elongation. Furthermore, the promoter region of *GhKCS1b_Dt* contains elements related to the BES1 binding site G box (Yang et al., 2023). This finding invites further investigation into the regulatory effects of *GhBES1.4* on *GhKCS1b_Dt* to confirm its role in modulating fiber elongation through the BR and VLCFA pathways.

In conclusion, we developed a comprehensive CMRN dataset encompassing various tissues throughout the entire growth cycle of upland cotton and systematically investigated metabolic and transcriptional changes in the BR-deficient mutant *pag1* compared to ZM24. Our study revealed tissue-specific patterns of metabolite accumulation and gene expression across different developmental stages. Using the CMRN dataset, we identified differentially accumulated metabolites within the VLCFA synthesis pathway, leading to the identification of a key DEG, *GhKCS1b_Dt*, which positively regulates fiber elongation in cotton. The CMRN dataset can be used not only to identify and elucidate key regulatory genes and networks involved in BR-related metabolism but also to identify metabolites and key genes related to metabolism and signal transduction in fiber development, providing valuable genetic resources for cotton breeding.

## Methods

### Plant materials and sample preparation

This study utilized two cotton materials, the *G. hirsutum* cultivar ZM24 and the BR-deficient mutant *pag1* (Figure 1; Supplemental Table 9). Seeds of both ZM24 and *pag1* cotton were grown in a greenhouse under a 14/10 h light/dark cycle. Daytime temperatures ranged from 28°C to 34°C, while night-time temperatures varied between 24°C and 27°C. Root samples were collected on the fifth and 15th days post-seeding (root 5 and root 15, respectively). Stem samples were harvested on the 15th, 35th, and 70th days post-seeding (stem 15, stem 35, stem 70). Flowers were collected on the 70th day post-seeding (flower 70), and leaf samples were collected on the 15th, 35th, and 70th days after the emergence of the first true leaf (leaf 15, leaf 35, leaf 70). Ovule and fiber tissues were extracted at 5, 10, and 20 DPA (ovule/fiber 5, ovule/fiber 10, ovule/fiber 20). All samples were immediately harvested and frozen in liquid nitrogen. Metabolomic and transcriptomic analyses were performed using three biological replicates, each comprising a pooled sample from at least three individual plants.

### Metabolite extraction

Tissue powder from each sample was prepared by grinding approximately 0.4 g of frozen tissue on ice. The metabolomic analysis was conducted using a liquid chromatography–mass spectrometry (LC/MS) system, which included a Waters Acquity I-Class PLUS ultra-high-performance LC (UHPLC) coupled with a Waters Xevo G2-XS quadrupole time-of-flight (QTOF) high-resolution mass spectrometer. The metabolomic sequencing was performed at Biomarker Technologies using the UHPLC–QTOF–MS analysis method described by Wang et al. (Wang et al., 2016). The advantage of UHPLC–QTOF–MS lies in its ability to capture all metabolite signals through high-resolution MS, enabling the discovery of unknown metabolites and the development of metabolite databases (Yang et al., 2022).

### Metabolite data preprocessing, annotation, and analysis

Raw data were extracted using MassLynx v.4.2 and imported into Progenesis QI for peak picking, alignment, and further analysis. Metabolites were identified using the online METLIN database (https://ngdc.cncb.ac.cn/databasecommons/database/) embedded in Progenesis QI and a custom library from Biomarker Company. Mass accuracy was ensured by matching to theoretical fragment identification, with all masses deviating within 100 ppm. Normalization was performed using peak area normalization across samples within the same tissue type. PCA and Spearman correlation analysis were used to assess sample repeatability. Identified compounds were categorized and analyzed for pathway involvement using KEGG, HMDB, and LIPID MAPS databases.

Fold changes were calculated for corresponding tissues of the two cotton materials, and a Student’s *t*-test was used to determine the significance of differences between each compound. Operational partial least squares discriminant analysis (OPLS–DA) modeling was conducted using the R package “ropls,” with model validation achieved through 200 permutation tests. Multiple cross-validation techniques were applied to calculate variable importance plots (VIP) values. Differentially expressed metabolites were identified based on the following criteria: a fold change greater than 1, a *P* value less than 0.01, and a VIP value greater than 1 in the OPLS-DA model.

### RNA library construction and sequencing

RNA was extracted from various tissue samples using an RNA Purification Kit and then used to generate RNA sequencing libraries with the NEBNext Ultra RNA Library Prep Kit. RNA sequencing was performed on an Illumina NovaSeq 6000 system using 150 base pair paired-end reads.

### Identification and functional annotation of DEGs

Clean reads were mapped to the *G. hirsutum* ZM24 reference genome (Yang et al., 2019) using HISAT2 (Kim et al., 2015). Gene expression levels were quantified using the fragments per kilobase of transcript per million mapped reads method (Trapnell et al., 2010). DEGs were identified using DESeq2, based on a log2 fold change of >1 and an adjusted *P* value of <0.05 (Wang et al., 2010). Gene Ontology and KEGG pathway analyses were performed using the R package "clusterProfiler" (Wu et al., 2021b).

### Co-expression network construction and screening of hub genes

A weighted gene co-expression network was constructed using the R package “WGCNA” (Langfelder and Horvath, 2008), with fragments per kilobase of transcript per million mapped reads values of DEGs as input. The network was built with default settings, except the soft-power parameter was set to 9, the minModuleSize parameter to 30, and the CutHeight parameter to 0.2. The contents of six metabolites in the BR pathway were used as phenotypes to link genes and identify aggregated modules, which were visualized with distinct colors. Hub genes were selected based on their eigengene connectivity (KME) values and the high weights of the modules.

### Vector construction, cotton transformation, and phenotypic analysis

The coding sequences of *GhKCS1b_Dt* were obtained from CottonGen (https://www.cottongen.org/). Specific primers listed in Supplemental Table 10 were used to amplify the *GhKCS1b_Dt* gene from a ZM24 cDNA library. The amplified fragment was inserted into the pCAMBIA-2300 vector, driven by the 35S promoter, to construct the *GhKCS1b_Dt* OE vector. The *GhKCS1b_Dt* RNAi vector was constructed by amplifying the *GhKCS1b_Dt*-RNAi sequence from ZM24 cDNA and subsequently cloning it into the pBI121 vector.

All constructs for OE and RNAi were introduced into the *Agrobacterium* strain LBA4404. ZM24 seeds were sterilized and cultured in a light-deprived chamber at 30°C for 6 days (Ge et al., 2023). The seeds were then cut into 5 mm segments and submerged in an *Agrobacterium tumefaciens* suspension (optical density = 0.2–0.4) for 15 min. Following callus induction, proliferation, embryogenic callus induction, embryo differentiation, and plantlet regeneration, the putative transgenic plants were transferred to pots and cultivated in a greenhouse under 14 h of light and 10 h of darkness at 25°C. The expression levels of *GhKCS1b_Dt* in OE and RNAi lines were detected using qRT–PCR, with the cotton *histone3* gene (GenBank: AF024716) serving as an internal control.

Phenotypic analysis of fibers was conducted on 30 bolls from each line (*GhKCS1b_Dt*-OE, *GhKCS1b_Dt*-RNAi, and wild-type ZM24).

### qRT–PCR analysis

The qRT–PCR was conducted using TransStart Top Green qPCR SuperMix (TransGen Biotech, Beijing, China) on an Applied Biosystems 7500 Fast Real-Time PCR System. The PCR protocol included an initial denaturation at 95°C for 5 min, followed by 40 cycles of denaturation at 95°C for 15 s, annealing at 60°C for 15 s, and extension at 72°C for 20 s. The *GhHistone3* gene (GenBank: AF024716) served as an internal reference. Relative gene expression levels were calculated using the 2^−ΔΔCT^ method (Livak and Schmittgen, 2001).

### Determination of BR content via LC–MS/MS

Two-week-old leaves of ZM24 and *pag1* were harvested, immediately flash-frozen in liquid nitrogen, and ground into a fine powder. For extraction, 50 mg of each sample was combined with 1 ml of HPLC-grade acetonitrile and spiked with 10 μl of an internal standard solution (10 ng/ml). The mixtures were vortexed for 10 min and then centrifuged at 12 000 rpm for 10 min at 4°C. The supernatant was transferred to 2 ml amber glass vials, to which 200 μl of 4-(N, N-dimethylamino) phenylboronic acid was added. The mixtures were vortexed and incubated at 75°C for 1 h before drying under a nitrogen gas stream. The residue was reconstituted in 100 μl of acetonitrile and filtered through a 0.22 μm membrane for LC–tandem MS (MS/MS) analysis (Ding et al., 2016; Yu et al., 2018). The analysis was performed on a UPLC–electrospray ionization–MS/MS system (UPLC, ExionLC AD; MS, Applied Biosystems 6500 Triple Quadrupole) with a Waters ACQUITY UPLC HSS T3 C18 column using a gradient solvent system and specific electrospray ionization conditions to detect BRs through scheduled multiple reaction monitoring (Xin et al., 2013; Ding et al., 2014). BL (CAS: 72962-43-7) and CS (CAS: 80736-41-0) standards were sourced from Olechemin (purity >98%). BR content analysis was conducted by MetWare (http://www.metware.cn/) on the AB Sciex QTRAP 6500 LC–MS/MS platform.

### Fatty acid extraction and gas chromatography–MS analysis

Prior to fatty acid extraction, 0.2 g of ZM24, *GhKCS1b_Dt*-OE, and *GhKCS1b_Dt*-RNAi fibers (10 DPA) were ground in liquid nitrogen. The powdered fiber was then mixed with a 5% sulfuric acid/methanol solution (v/v) and 10 μl of butylated hydroxytoluene in methanol, followed by 1 min of vortexing. The samples were incubated at 90°C for 30 min and then cooled to room temperature. After adding 2 ml of hexane and 10 ml of saturated sodium chloride solution, the mixture was vortexed and allowed to settle. The supernatant was collected for analysis. Fatty acid methyl esters were identified using the Supelco 37 FAME Mix standard and analyzed by gas chromatography with an HP-5 capillary column (30 m × 0.25 mm × 0.25 μm, Agilent 7890-5977 system). Fatty acid content was quantified using an external standard method and reported in μg/g fresh weight (Dursun and Guler, 2021).

### *In vitro* ovule culture

Ovules from ZM24 and *GhKCS1b_Dt* cotton plants at 1 DPA were sterilized by immersing them in 75% ethanol for 5 min, followed by a brief soak in 95% ethanol for 2–3 s. The procedure included five rinses with distilled water to ensure thorough cleaning. The sterilized ovules were then transferred into a liquid BT medium. C24:0 (CAS: 557-59-5) (5 μM/l) and the VLCFA inhibitor 2-chloro-N-(ethoxymethyl)-N-(2-ethyl-6-methyl-phenyl)-acetamide (CAS: 32 428-71-0) (2 μM/l) were dissolved in anhydrous ethanol before being added to the BT medium (Qin et al., 2007). Subsequently, the ZM24 and *GhKCS1b_Dt* transgenic cotton ovules were immersed in the BT medium containing C24:0 and 2-chloro-N-(ethoxymethyl)-N-(2-ethyl-6-methyl-phenyl)-acetamide. The ovules were cultured in the dark at 30°C for 14 days, after which the fiber length was measured.

### Statistical analysis

Gene expression levels and metabolite contents were normalized using the *Z*-score normalization method. Hierarchical clustering and PCA were performed using the R packages “pheatmap” and “ggplot2.” Co-regulation analysis was conducted on samples from 30 different tissues of two materials at various developmental stages using the k-means clustering method within the MeV v.4.9 software (Gasch and Eisen, 2002). All qRT–PCR and phenotypic data were analyzed using one-way ANOVA or a two-tailed Student’s *t*-test with GraphPad Prism. Values are presented as mean ± SD.

## Data and code availability

All datasets generated for this study are included in the article/supplemental information. The detailed datasets can be provided by the corresponding author on a reasonable request.

## Funding

This study was supported by the 10.13039/501100001809National Natural Science Foundation of China (grants 32441062, 32360509, 32301888, and 32350410409); the National Key Laboratory of Cotton Bio-breeding and Integrated Utilization (CBIU2024004); the Xinjiang Science and Technological Program (the Tianshan Talent Training Program [grant 2022TSYCCX0087], the Key Research and Development Program of Xinjiang [grant 2022B02052], and the Natural Science Foundation of Xinjiang Uygur Autonomous Region [grants 2022D01E08 and 2024D01A150]); the Xinjiang Science and Technology Major Project of China (grant 2024A02002) and the 10.13039/501100006407Natural Science Foundation of Henan Province (grant 232300421253).

## Acknowledgments

No conflict of interest is declared. We thank Prof. Yang Zhang from Sichuan University for constructive suggestions.

## Author contributions

F.L. and Z.Y. conceived and designed the research. Z.L., L.F., S.S., and G.Q. performed the experiments. Z.L. and E.C. contributed materials and assisted in phenotyping. Z.L., J.H., and L.F. analyzed the data. Z.L., L.F., S.S., and G.Q. wrote the manuscript. All authors have read and approved the content of this article.

## Supplemental information

Data S1. Supplemental Tables 1–10

Document S2. Article plus supplemental information

## Footnotes

Supplemental information is available at *Plant Communications Online*.
