# Supplementary material for: Cotton metabolism regulatory network: Unraveling key genes and pathways in fiber development and growth regulation
Source: Plant Commun. 2024 Dec 12;6(3):101221. doi: 10.1016/j.xplc.2024.101221 (PMC11956105; doi:10.1016/j.xplc.2024.101221)
Supplement: Document S1. Supplemental Figures 1–9 [file mmc1.pdf]

Plant Communications, Volume *6*

Supplemental information

Cotton metabolism regulatory network: Unraveling key genes and pathways in fiber development and growth regulation

Zhao Liu, Liqiang Fan, Sheng Shu, Ghulam Qanmber, Eryong Chen, Jinquan Huang, Fuguang Li, and Zuoren Yang

Supplemental Information

Cotton Metabolism Regulatory Network: unraveling key genes and pathways in fiber development and growth regulation

Zhao Liu[1,3†], Liqiang Fan[2,3†], Sheng Shu[1†], Ghulam Qanmber[1], Eryong Chen[4], Jinquan Huang[5], Fuguang Li[1,2,3*], Zuoren Yang[1,2,3*]

[1] Zhengzhou Research Base, State Key Laboratory of Cotton Bio-breeding and Integrated Utilization, School of Agricultural Sciences, Zhengzhou University, Zhengzhou 450001, Henan, China

[2] State Key Laboratory of Cotton Bio-breeding and Integrated Utilization, Institute of Cotton Research, Chinese Academy of Agricultural Sciences, Anyang 455000, Henan, China

[3] Institute of Western Agriculture, the Chinese Academy of Agricultural Sciences, Changji 831100, Xinjiang, China

[4] Henan Engineering Research Center of Crop Genome Editing, School of Agriculture, Henan Institute of Science and Technology, Xinxiang 453000, Henan, China

[5] National Key Laboratory of Plant Molecular Genetics, CAS Center for Excellence in Molecular Plant Sciences, Shanghai Institute of Plant Physiology and Ecology, Chinese Academy of Sciences, Shanghai 200032, China

Corresponding authors:

Fuguang Li: aylifug@caas.cn

Zuoren Yang: yangzuoren@caas.cn

Authors information:

Zhao Liu: liuzhaocaas@zzu.edu.cn

Liqiang Fan: fanliqiang@caas.cn

Sheng Shu: shusheng0609@163.com

Ghulam Qanmber: gqkhan12@zzu.edu.cn

Eryong Chen: lovelycheneryong@163.com

Jinquan Huang: huangjinquan@cemps.ac.cn
Fuguang Li: aylifug@caas.cn
Zuoren Yang: yangzuoren@caas.cn

† These authors have contributed equally to this work.

Supplementary figures

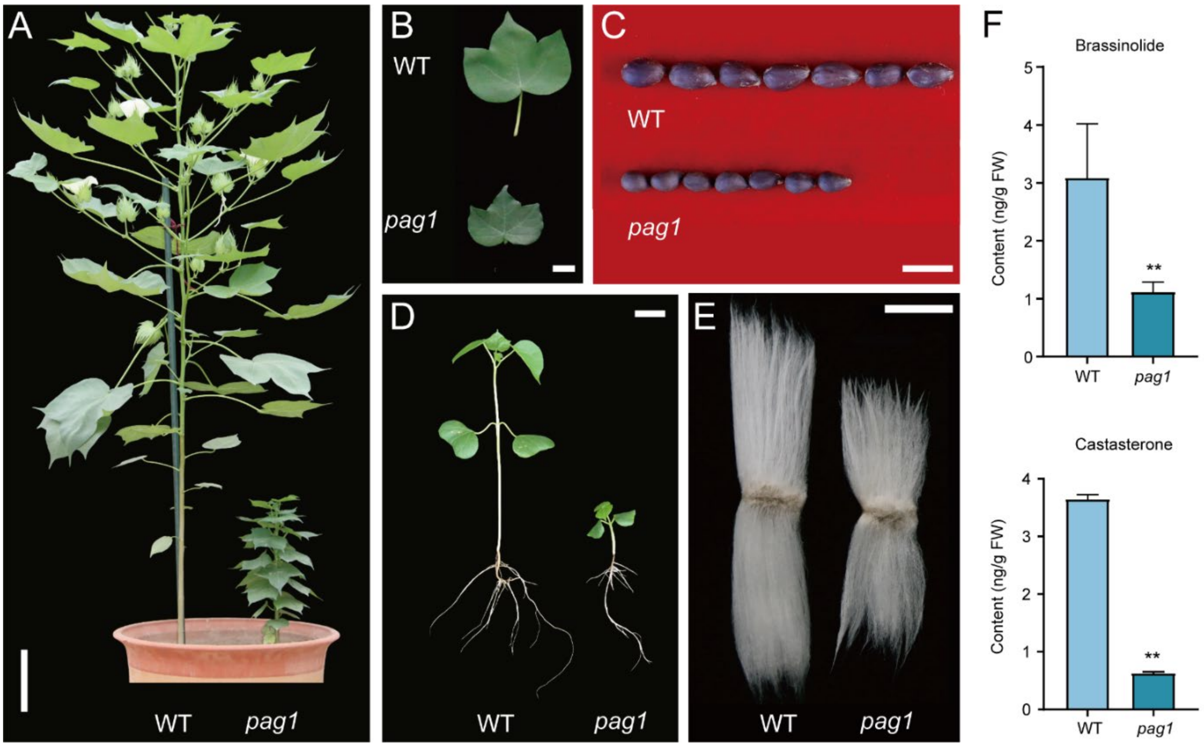

Supplemental Figure 1. Phenotypes of *pag1* mutant. (A) Phenotypes of wild-type (WT) and *pag1* mutant. Bar=20 cm. (B) Phenotypes of leaves. Bar=1 cm. (C) Phenotypes of seeds. Bar=1 cm. (D) Phenotypes of plants in the seeding stage. Bar=1 cm. (E) Phenotypes of mature fibers. Bar=1 cm. (F) BR contents (brassinolide and castasterone) in WT and *pag1* cotton.

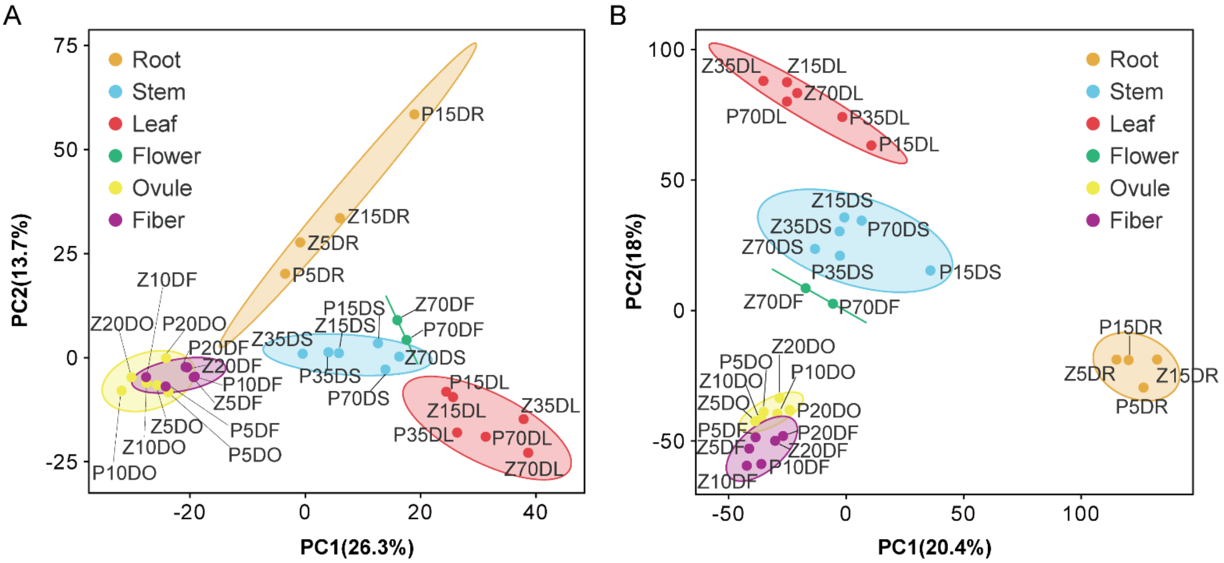

Supplemental Figure 2. PCA of metabolome (A) and transcriptome (B) data in seven clusters from the 90 cotton samples.

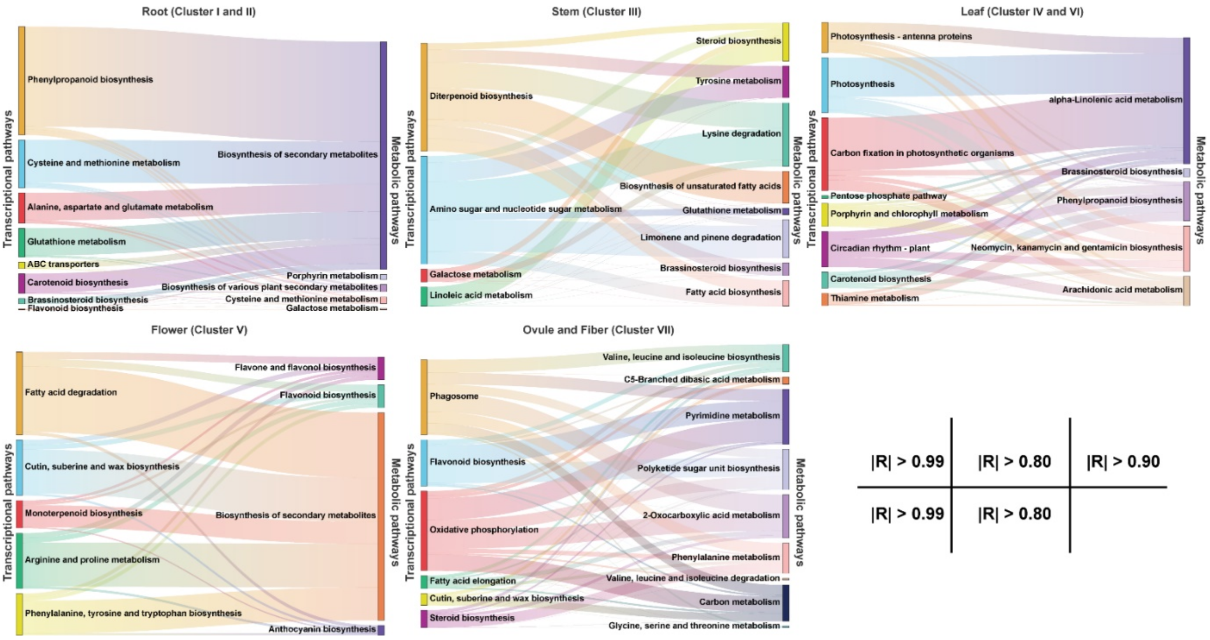

Supplemental Figure 3. Correlation result of transcriptional pathways and metabolic pathways for five types of tissues of *pag1* and ZM24. Thresholds were set based on relevance in each tissue. Genes and metabolites with R > 0.99 were screened in roots and flowers; genes and metabolites with R > 0.90 were screened in leaves; and genes and metabolites with R > 0.80 were screened in stems, ovules, and fibers.

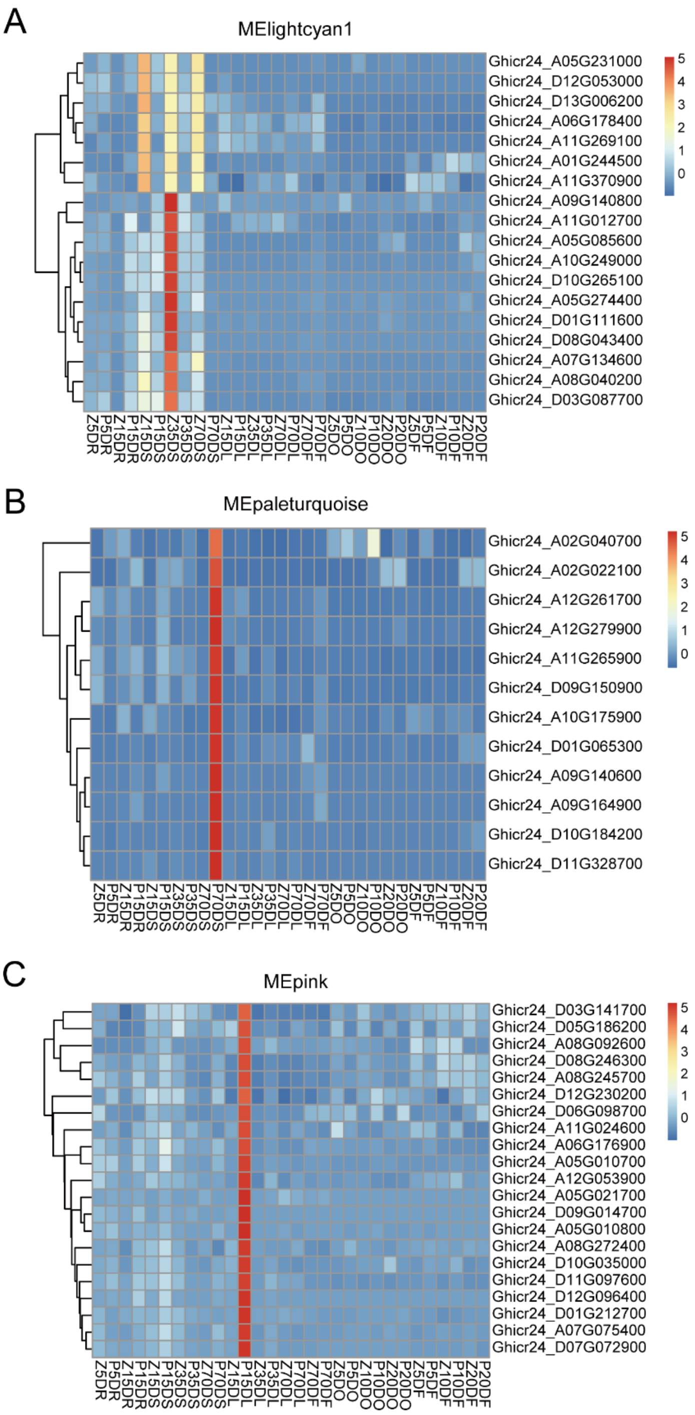

Supplemental Figure 4. Heatmap showing the expression of hub genes in the ME lightcyan 1 module, ME paleturquoise module, and ME pink module.

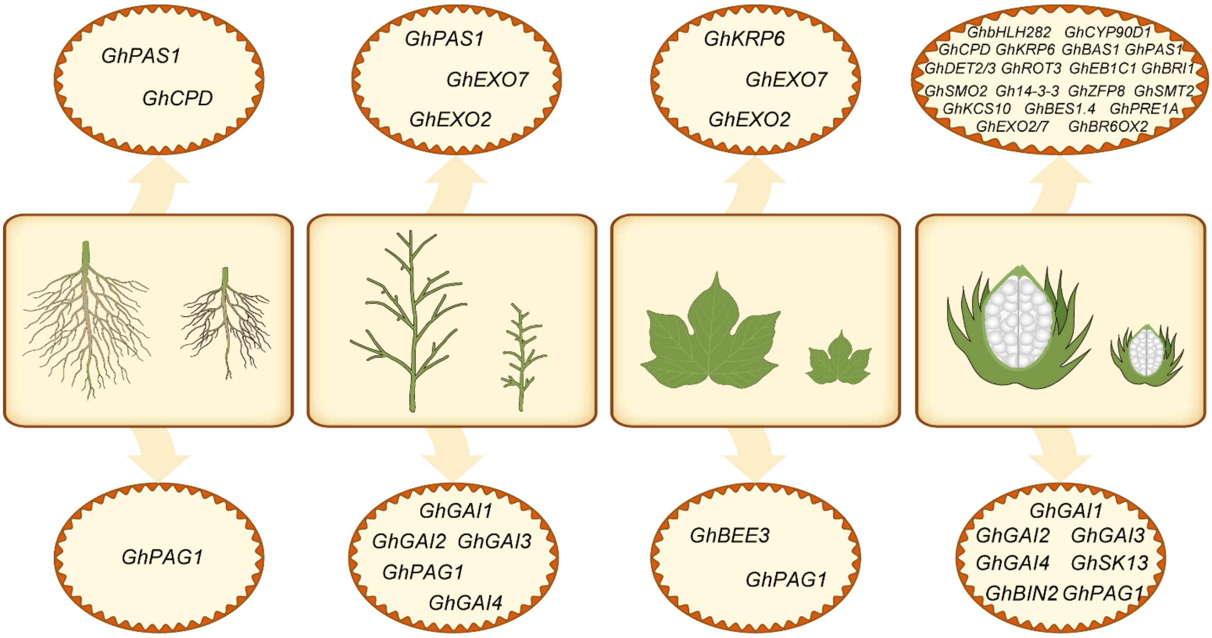

Supplemental Figure 5. Genes related to the BR synthesis and signaling pathways in different tissues of cotton. Upper panel: Genes associated with BR synthesis and signal transduction positively regulate different tissues of cotton. Middle panel: Morphological demonstration of cotton roots, stems, leaves, ovules, and fibers under normal and BR-deficient conditions in BR. Lower panel: Genes associated with BR synthesis and signal transduction negatively regulate different tissues of cotton.

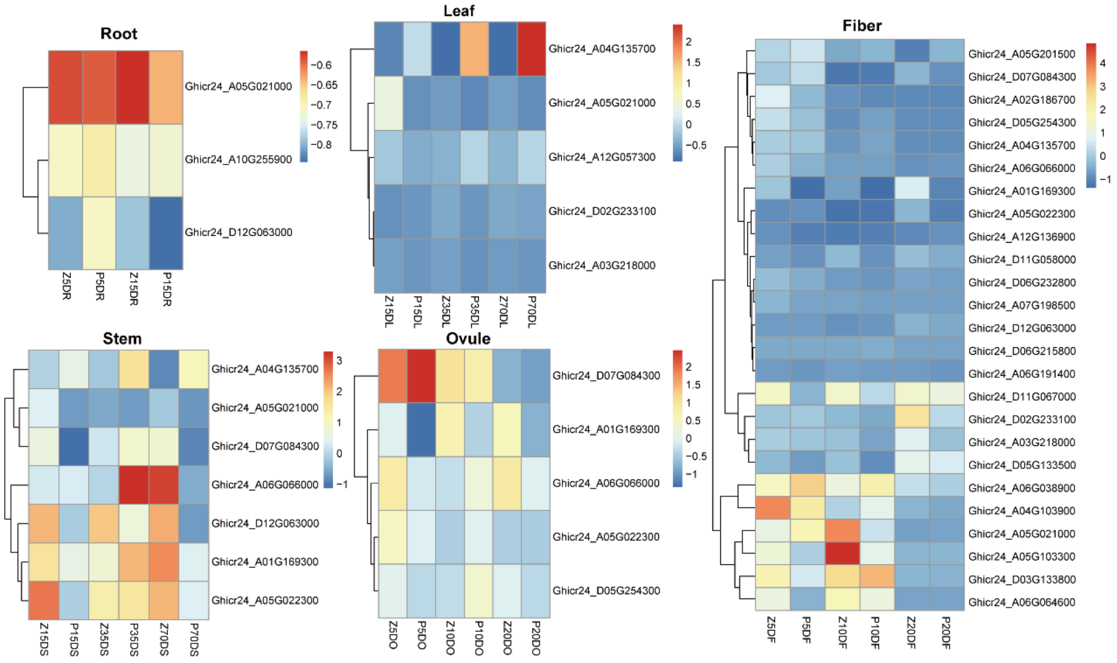

Supplemental Figure 6. Heatmap showing the expression of genes related to BR synthesis and signaling pathways in roots, stems, leaves, ovules, and fibers of ZM24 and *pag1* under normal and BR-deficient conditions.

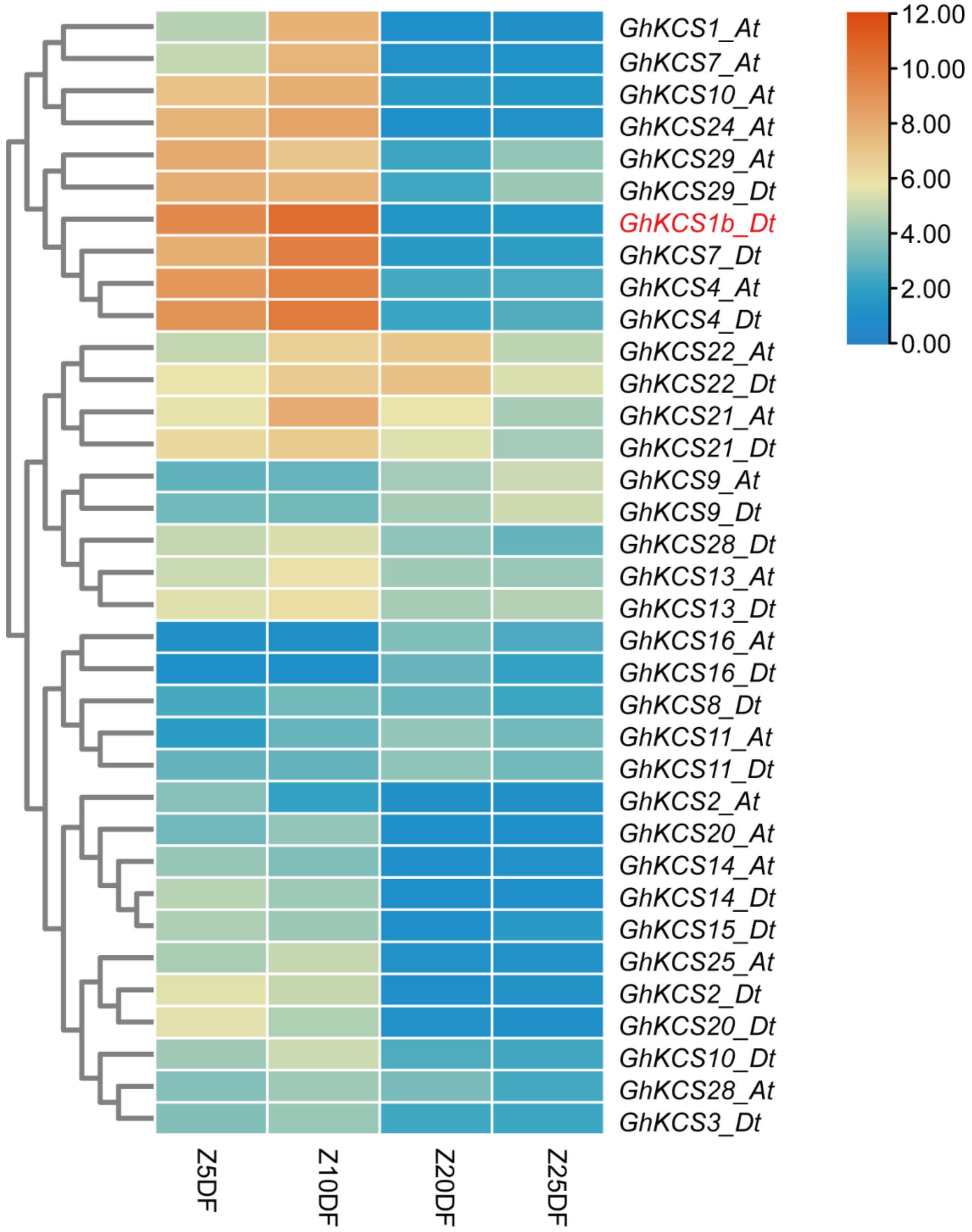

Supplemental Figure 7. Heatmap showing the expression patterns of fiber dominant *GhKCS* genes in ZM24 fiber at 5, 10, 20, and 25 DPA.

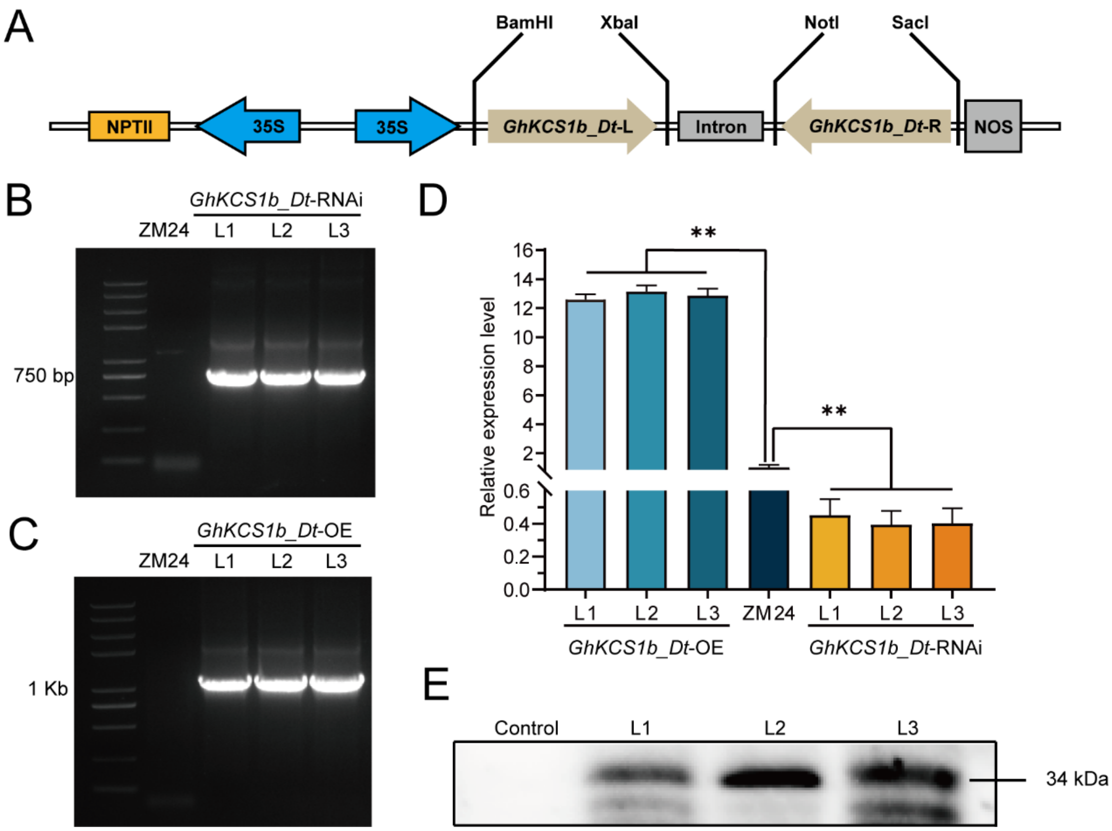

Supplemental Figure 8. Confirmation of *GhKCS1b_Dt* expression in overexpression and RNAi lines. Expression of *GhKCS1b_Dt* was confirmed in *GhKCS1b_Dt*-OE and *GhKCS1b_Dt*-RNAi lines at the DNA (A-C), RNA (D), and protein (E) levels. For qRT-PCR analysis, *Histone3* served as the internal control. Error bars represent SD for three independent experiments. The expression level in the ZM24 sample was normalized to 1.

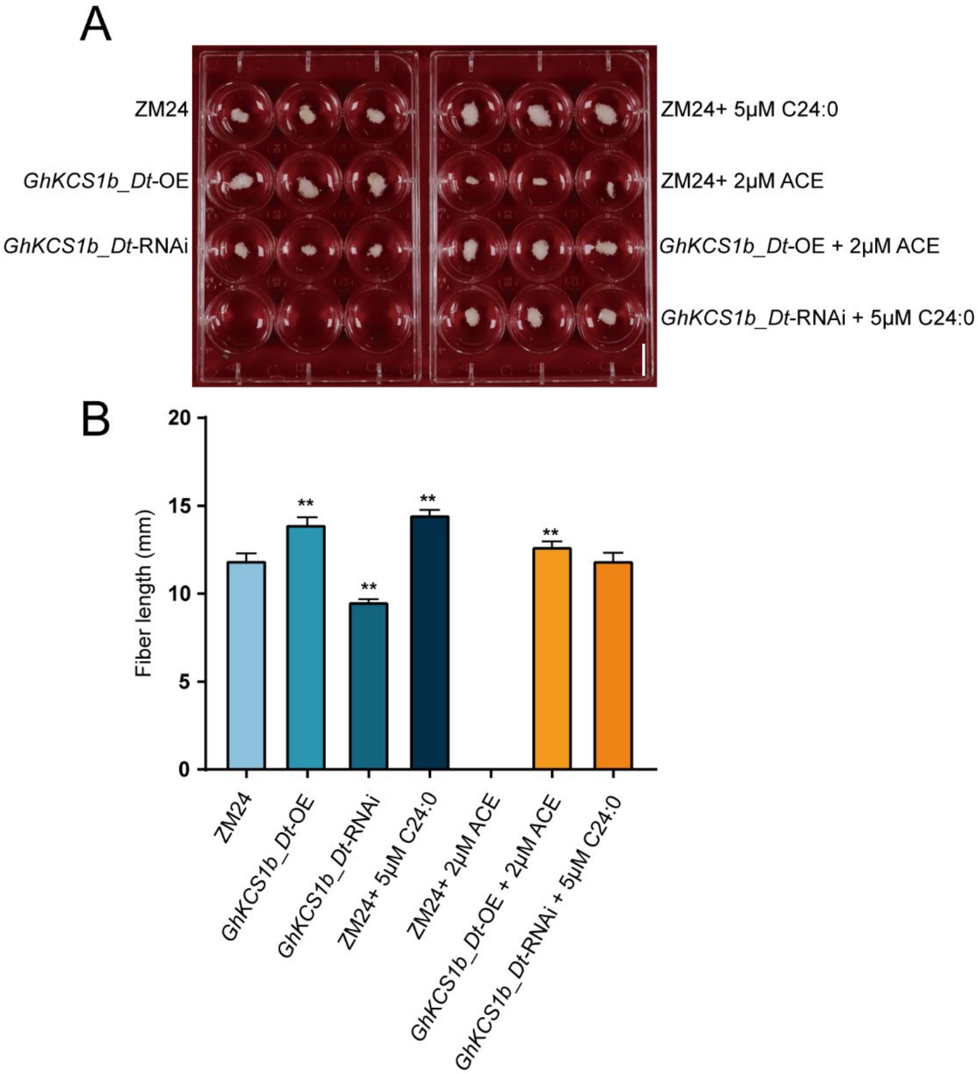

Supplemental Figure 9. C24:0 partially restores the fiber-shortening phenotype associated with *GhKCS1b_Dt*. (A) Characteristics of ZM24 ovules, *GhKCS1b_Dt*-OE, and *GhKCS1b_Dt*-RNAi (harvested at 1 DPA) after 14 days of culture in media supplemented with or without C24:0 (5 μM) or ACE (2 μM). (B) Final fiber lengths were measured at the end of the 14-day culture period. Scale bar = 1 cm.
